# Acriflavine, a HIF-1 inhibitor, preserves vision in an experimental autoimmune encephalomyelitis model of optic neuritis

**DOI:** 10.3389/fimmu.2023.1271118

**Published:** 2023-10-23

**Authors:** Jeffrey J. Anders, Benjamin W. Elwood, Randy H. Kardon, Oliver W. Gramlich

**Affiliations:** ^1^Department of Ophthalmology and Visual Science, The University of Iowa, Iowa City, IA, United States; ^2^Center for the Prevention and Treatment of Visual Loss, Iowa City Veterans Affairs (VA) Health Care System, Iowa City, IA, United States; ^3^Department of Neuroscience and Pharmacology, The University of Iowa, Iowa City, IA, United States

**Keywords:** EAE, optic neuritis, multiple sclerosis, retinal ganglion cells, acriflavine, HIF-1 inhibition, unfolded protein response

## Abstract

**Introduction:**

Optic neuritis (ON) is often an early sign of multiple sclerosis (MS), and recent studies show a link between HIF-1 pathway activation and inflammation. This study aimed to determine if inhibition of the HIF-1 pathway using the HIF-1a antagonist acriflavine (ACF) can reduce clinical progression and rescue the ocular phenotype in an experimental autoimmune encephalomyelitis (EAE) ON model.

**Methods:**

EAE-related ON was induced in 60 female C57BL/6J mice by immunization with MOG33-55, and 20 EAE mice received daily systemic injections of ACF at 5 mg/kg. Changes in the visual function and structure of ACF-treated EAE mice were compared to those of placebo-injected EAE mice and naïve control mice.

**Results:**

ACF treatment improved motor–sensory impairment along with preserving visual acuity and optic nerve function. Analysis of retinal ganglion cell complex alsoshowed preserved thickness correlating with increased survival of retinal ganglion cells and their axons. Optic nerve cell infiltration and magnitude of demyelination were decreased in ACF-treated EAE mice. Subsequent in vitro studies revealed improvements not only attributed to the inhibition of HIF-1 butalso to previously unappreciated interaction with the eIF2a/ATF4 axis in the unfolded protein response pathway.

**Discussion:**

This study suggests that ACF treatment is effective in an animal model of MS via its pleiotropic effects on the inhibition of HIF-1 and UPR signaling, and it may be a viable approach to promote rehabilitation in MS.

## 1 Introduction

The term multiple sclerosis (MS) summarizes a heterogenous and multifactorial immune-driven disorder of the central nervous system (CNS). The major hallmark of MS is the degeneration of oligodendrocytes leading to demyelination, which is associated with a variable degree of axonal and neuronal loss (1, 2). Involvement of the HIF-1 pathway has been associated with MS as a potential driver for inflammatory demyelination (3). Histopathological studies on MS donor brains show the presence of HIF-1α in type III lesions. These type III lesions are characterized by low inflammation defined as distal “dying back” oligodendrocyte degeneration (4), and subsequent studies suggest that hypoxia accompanied by the generation of reactive oxygen species and nitric oxide may be early triggers of inflammation in MS. These hypoxia-like conditions are perpetuated by other MS pathologies such as reduced CNS blood flow, blood–brain barrier disruption, and vascular inflammation, consequently causing low oxygen levels at lesion sites that already have an increased energy demand. Furthermore, the sum of these hypoxia factors leads to mitochondrial dysfunction, exacerbating a potential metabolic crisis as an important pathologic mechanism in MS and in the experimental autoimmune encephalomyelitis (EAE) animal model [reviewed in (5, 6)]. A more recent study reports the upregulation of genes related to hypoxia, including for *Hif1-α*, in the choroid plexus of MS patients. Importantly, levels of hypoxia-responsive secreted peptides in the cerebrospinal fluid correlate with the grade of disability in the investigated MS cohort (7). Relevant to the autoimmune pathology of MS, HIF-1 signaling plays an important role in regulation of the immune system. HIF-1 expression is upregulated in immune cells under normoxic conditions in response to stimulation through Toll-like receptors or T cell receptors and CD28 signaling via the mammalian target of rapamycin (mTOR) pathway activation. The presence of HIF-1α affects the fate and function of T cell subsets, particularly T helper 17 (Th17) cells and regulatory T cells. For example, HIF-1α is directly involved in Th17 T cell differentiation via direct transcriptional activation of the retinoic acid receptor related to the orphan receptor γT (RORγT) and recruitment of the co-stimulatory p300 to the IL-17 promoter (8). Furthermore, HIF-1 facilitates Foxp3 protein degradation and thus inhibits regulatory T cell (Treg) differentiation. Knockout of *Hif1-α* halts Th17 differentiation and enhances Treg development (reviewed in (9, 10)). This is relevant to MS because Th17 cells play an important role in MS patients and in mounting a MS phenotype in the EAE model (11). It has also been shown in the EAE model that T cell-specific *Hif1-α* knockout results in the protection of mice from demyelination. The resistance to EAE in *Hif1-α* knockout mice is related to the suppression of Th17 cell development in favor of Treg differentiation (12). Another study demonstrated that altering the Th17/Treg balance using the drug metformin to modulate the mTOR/AMP-activated protein kinase (AMPK)/HIF-1 axis ameliorated the development of EAE (13).

Inflammation of the optic nerve, herein referred to as optic neuritis (ON), is a common cause of vision loss, particularly in demyelination disorders (14). ON is the initial presenting symptom in one in four MS cases, and up to 35% of MS patients experience recurrent ON during the course of their disease (15–17). While recovery of high-contrast visual acuity occurs in 90% of affected eyes, patients with MS complain of significant decreases in contrast sensitivity, impaired color vision and motion perception, visual field disturbances, and stereopsis (18). Functional impairment of the visual system in MS is further evident as a delay in optic nerve conduction speed in visual evoked potential (VEP) recordings and also a decrease in the pattern electroretinogram amplitude (19, 20). Importantly, current studies have demonstrated that VEP latency correlates with optic nerve demyelination and remyelination (21), and VEP recordings have been recently established as a valuable tool for studying neurodegeneration and testing neuroprotective drugs in the context of animal models of demyelination (22, 23). VEP recordings also have potential prognostic value for subsequent retinal ganglion cell (RGC) loss in MS (24). Thinning of the retinal nerve fiber layer (RNFL) and RGC layer as measured by optical coherence tomography (OCT) imaging in MS patients over time are reliable indicators of structural RGC degeneration (25, 26) Quantification of the macular ganglion cell layer/inner plexiform layer (IPL) thickness is currently the most used OCT marker to determine retinal atrophy in MS (27, 28). In animal models of MS, OCT imaging has been implemented to increase the translational rigor, and a recent study has demonstrated a correlation between changes in retinal layers and RGC loss (29). Thus, the visual pathway is most susceptible to disease activity, and neuro-ophthalmic measurements have been frequently used as primary and secondary outcome parameters in clinical MS trials (30, 31). RGCs are essential retinal neurons responsible for transmitting the electrical signal from the retina via myelinated optic nerve axons to the lateral geniculate nucleus and, from there, to the visual cortex in the brain (32). RGCs are especially vulnerable to inflammation (33), sensitive to oxygen deprivation and HIF-1 signaling, and at risk for impaired energy metabolism in optic neuropathies (34). It is therefore conceivable that some aspects of pathologic changes of visual function and structure during and after MS-associated ON are similar to the HIF-1 pathology seen in MS brains.

Clinical and preclinical studies (7, 35), including ours (36), indicate the involvement of the HIF-1 signaling pathway in MS, and together, these findings suggest that inhibition of HIF-1 may provide a new strategy for the treatment of MS. In this study, we utilized two different HIF-1 inhibitors, acriflavine (ACF) and echinomycin (Echi), to determine immunomodulatory and restorative effects in MOG_33-55_-induced EAE. This EAE model is particularly relevant to visual impairment in MS because it consists of a well-established ON phenotype. ON in MOG-induced EAE is quantified by means of highly translational visual systems metrices (visual acuity, OCT, electrophysiology) and characteristic retinal and optic nerve histopathology (29, 37–39). ACF, an FDA-approved compound, directly binds to the HIF-1α subunit, which inhibits HIF-1 dimerization and subsequently blocks its transcriptional activity (40). ACF also interferes with the unfolded protein response (UPR) pathway via inhibition of eukaryotic translation initiation factor 2 (eIF2a) phosphorylation, causing a downregulation of activating transcription factor 4 (ATF4) translation and signal transducer and activator of transcription 5 (Stat5) in a HIF-1-independent manner. These properties further warrant ACF as a potent inhibitor of UPR, as demonstrated in the most recent cancer therapies (41, 42). In addition, ACF has a favorable pharmacokinetic profile in rodents (43) and humans (44), with fast plasma and systemic clearance and topical application, which showed its bioavailability in CNS tissue and the retina (45, 46). Echinomycin specifically inhibits binding of the HIF-1 dimer to the hypoxia response element sequence contained in the vascular endothelial growth factor (Vegf) promoter (47). Thus, echinomycin’s mechanism of action more selectively targets the VEGF-related pathway.

In this study, we found that ACF administration, but not Echi injections, was effective in reducing motor–sensory impairments in EAE mice. Systemic ACF treatment of EAE animals also mitigated the decline in visual function and structure, including preservation of RGC density and optic nerve axon numbers. Additionally, we found evidence that ACF enhances the electrical function of RGC, suggesting ACF may facilitate potential remyelinating or myelin-preserving effects. Lastly, we investigated whether this rescue effect is also mediated by ACF immunomodulatory properties in a dose-dependent manner in *in vitro* immunoassays.

## 2 Materials and methods

### 2.1 EAE model

**Figure 1:**
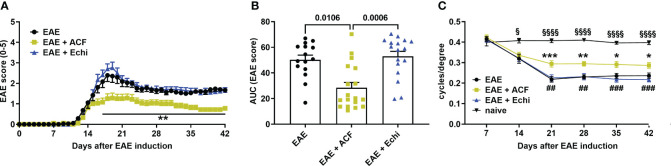
Time course of motor–sensory impairment in MOG-induced female EAE mice. **(A)** EAE scoring over time demonstrated a milder progression of EAE mice having received ACF (EAE+ACF, n=18, green) at day of EAE induction when compared to untreated (EAE, n=15, black) and Echi- treated EAE animals (EAE+Echi, n=17, blue). **(B)** Area-under-curve (AUC) analysis confirmed partial protection from severe decline in motor–sensory function in ACF-treated EAE mice. Data are given as group mean ± SEM, and p-values were calculated using two-way ANOVA with Tukey *post hoc* test in **(A)** and Kruskal–Wallis analysis in **(B)**. **(C)** Measurement of visual acuity by means of changes in cycles per degree confirmed preservation of function in ACF-treated EAE mice when compared to both EAE mice and the Echi-treated EAE group. Of note, all EAE mice demonstrated a decline in visual acuity compared to naïve control mice (n=10), and statistical differences between the naïve cohort and EAE-induced animals are displayed as § p<0.05 and §§§§ p<0.0001. The graphs in A and C display the pooled mean ± SEM per group, and the statistical difference between the EAE+ACF cohort and the untreated EAE group is indicated as *p<0.05, **p<0.01, and ***p<0.001 and as ## p<0.01 and ###p<0.001 compared to the EAE+Echi group. Analysis of visual acuity was performed using a mixed-effect model to account for repeated measurements of both eyes per sample.

All animal experiments were approved by the local IACUC and were conducted according to the ARVO Statement for the Use of Animals in Ophthalmic and Vision Research. EAE-ON was induced in eight-to-twelve-week-old female C57BL/6J mice (The Jackson Laboratories, Bar Harbor, ME), based on previously described methods (36, 48). Briefly, the mice were injected subcutaneously at two sites along the back with 250 μg of MOG_35–55_ (Sigma Aldrich, St. Louis, MO, USA) emulsified with complete Freund’s adjuvant (Sigma Aldrich) containing 2 mg/mL of mycobacterium tuberculosis (BD Difco, Franklin Lakes, NJ, USA). The animals were injected intraperitoneally with 400 ng of pertussis toxin (Sigma Aldrich, St. Louis, MO, USA) at day 0 and day 2. Motor–sensory function was assessed daily by a trained investigator who was masked to the study groups and treatment assignment using a 5-point scoring system with the following criteria: 0 = no symptoms, 0.5 = partial tail paralysis, 1 = tail paralysis, 1.5 = partial tail paralysis and waddling gait, 2 = tail paralysis and waddling gait, 2.5 = partial limb paralysis, 3 = paralysis of one limb, 3.5 = paralysis of one limb and partial paralysis of another, 4 = paralysis of two limbs, 4.5 = moribund state, and 5 = death (**Figure 1**).

### 2.2 Study design and HIF-1 inhibitors

Female C57BL/6J (B6) mice were housed under a 12 h light–dark cycle with access to water and food *ad libitum.* EAE-ON was induced in 60 mice using MOG immunization. Group assignment was randomly selected, and cohorts of 20 EAE mice were treated with 5 mg/kg ACF (EAE+ACF) intraperitoneally (IP) on day 0 and continued IP injections daily for the duration of the study. Another 20 EAE mice received 10 μg/kg echinomycin IP (EAE+Echi) on day 0 and were subsequently treated systemically every other day. Dosages were selected based on published reports demonstrating sufficient bioavailability (45, 49). The 20 EAE mice received an equivalent volume of the vehicle solution PBS (Gibco, Waltham, MA, USA) and served as a vehicle-injected control EAE group (EAE). Another 10 naïve, age-matched mice served as controls (naïve). Disease course progression was measured daily using a 5-point scoring system. Visual acuity was assessed weekly using optokinetic response (OKR). Subsequently, all animals underwent ocular coherence tomography (OCT) imaging at day 21 and at the end of the study, along with pattern visual evoked potential (pattern VEP) recordings prior to euthanasia. Assessment of outcome parameters was performed by trained personnel who were masked to the study groups (EAE and naïve) and treatment assignment (placebo vs. ACF vs. Echi treatment) to minimize potential bias. All animals were euthanized by CO_2_ inhalation followed by cervical dislocation and tissue harvest 42 days after EAE induction.

### 2.3 Visual acuity

Bilateral visual acuity was measured using the OptoDrum system (Striatech, Tübingen, Germany) as previously described (50). Awake mice were placed on a platform in a closed chamber with a virtual cylinder projecting stripes of varying spatial frequencies at 99.8% contrast. The mice were observed through a top-down camera, and reflexive head movements were detected by automated software. The highest spatial frequency as cycles/degree (c/d) detected by each eye reflected visual acuity.

### 2.4 OCT

**Figure 2:**
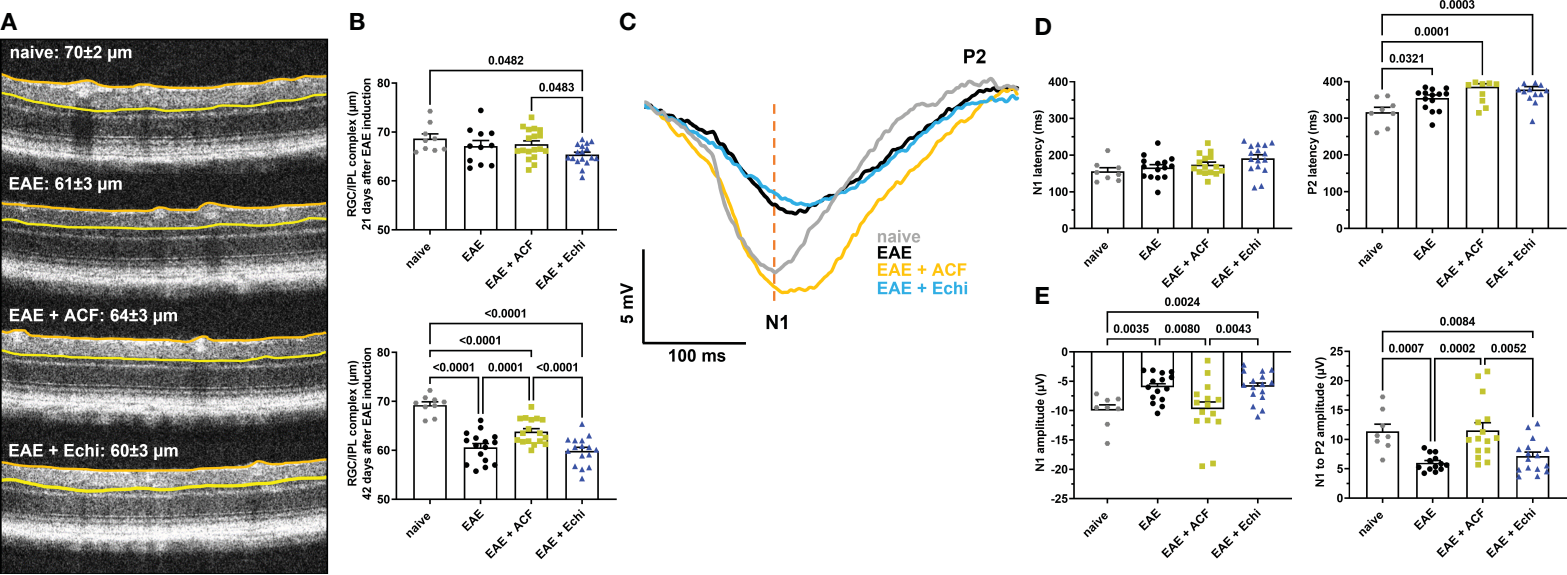
ACF treatment ameliorated degeneration of RGC structure and optic nerve function. **(A)** Representative OCT images demonstrate thinning of the RGC/ILP complex, outlined by the orange and yellow line, in treated and untreated EAE mice 42 days after immunization. **(B)** Measurements of the RGC/IPL complex over time revealed progressive decrease in thickness in all EAE groups, whereas ACF treatment significantly improved the outcome of RGC/IPL complex thinning. **(C)** Group-averaged pattern VEP trace overlays displayed significant changes in amplitudes, N1 latency, and P2 conduction speed. **(D)** Analysis of pattern VEP recordings revealed a significant delay of the P2 latency. **(E)** Similar to the RGC/IPL complex thinning, declines in the pattern VEP amplitudes were observed in the EAE and EAE+Echi groups. In contrast, ACF treatment facilitated preservation of the N1 and N1-to-P2 amplitudes in EAE mice. Points on graphs represent measurements of individual eyes. All data are given as mean ± SEM, and individual data points show the average value of both eyes per sample. Differences between groups were calculated using a mixed-effect model with Tukey *post hoc* test.

OCT was used to analyze the structure of the retina in 2% isoflurane (Baxter, Deerfield, IL, USA)-anesthetized mice using a SomnoSuite (Kent Scientific, Torrington, CT, USA). Prior to imaging, pupils were dilated using 1% tropicamide (Alcon Laboratories, Geneva, Switzerland). GenTeal gel (Alcon Laboratories, Alcon, Geneva, Switzerland) was placed on the corneal surface for preservation of corneal integrity. OCT images were obtained using an SD-OCT instrument (Bioptigen, Morrisville, NC, USA). Thickness of the RNFL, RGC, and the inner plexiform layer (IPL) was measured manually using calipers provided by the software. Briefly, thickness was measured between the borders of the RNFL from the inner limiting membrane and the outer borders of the IPL (referred to as the RGC/IPL complex) in the superior, inferior, nasal, and temporal quadrants 400 µM away from the optic nerve head (36; **Figure 2**). Data from eyes with indistinguishable retinal layer borders were excluded from future analysis.

### 2.5 Pattern VEP

Forty-two days after EAE induction, all animals underwent pattern VEP recordings on a Celeris platform (Diagnosys, Lowell, MA, USA). Briefly, after dark adaption overnight, the animals were anesthetized with intraperitoneal injection of ketamine (30 mg/kg, Mylan, Canonsburg, PA, USA), xylazine (5 mg/kg, Akorn Inc., Lake Forest, IL, USA), and acepromazine (2.3 mg/kg, Rattlesnake Drugs, Scottsdale, AZ, USA). Pupils were dilated using 1% tropicamide (Alcon Laboratories, Geneva, Switzerland), and GenTeal (Alcon Laboratories, Geneva, Switzerland) gel was used to keep the corneas moistened. The animals were kept on heating pads to maintain a constant body temperature of 37°C. Eyes were randomly selected, and 600 pattern VEP traces were recorded using a 2 Hz reversal vertical stripe pattern stimulus at a luminescence of 50 candela/m^2^ and 100% contrast. Amplitudes were measured in microvolts (µV) from the zero line to the N1 trough (N1 amplitude) and from the N1 trough to the P2 peak (N1 to P2 amplitude). Latencies in milliseconds (ms) were determined as the time from presentation of the stimulus to the N1 trough and the P2 peak. Data from eyes with flat or atypical pattern VEP waveforms or non-unique peak identification were excluded from the statistical analysis.

### 2.6 RGC density

The RNA-binding protein with multiple splicing (RBPMS) was used as a reliable marker for staining retinal ganglion cells in the mammalian retina (51). Eyes were fixed in 4% paraformaldehyde (PFA; Alfa Aesar, Ward Hill, MA, USA) for two hours before retinas were removed and stored in 0.3% Triton-X100/PBS at 4°C overnight. Retinas were blocked in 1%BSA/0.3% Triton-X100/1% DMSO/PBS for one hour at 4°C and then incubated with rabbit anti-RBPMS primary antibody (GTX118619 GeneTex, Irvine, CA, diluted 1:250 in 1%BSA/0.3% Triton-X100/1% DMSO/PBS) at 4°C overnight on a rocker platform. After washes in 0.3% Triton-X100/PBS, cells were visualized after incubation in goat anti-rabbit Alexa Fluor 488 secondary antibody (ab150077, Abcam, Cambridge, United Kingdom, diluted 1:200 in 1%BSA/0.3% Triton-X100/1% DMSO/PBS) for three hours at 4°C in the dark. After multiple washes in 0.3% Triton-X100/PBS, retinas were flat-mounted and cover-slipped using Aquamount (Epredia, Kalamazoo, Michigan, USA). Four images were taken at the mid-periphery regions of each retina at 20x magnification using a Zeiss Axioscope 5 (Carl Zeiss AG, Oberkochen, Germany). RGCs were counted semiautomatically using an ImageJ (National Institutes of Health, Bethesda, MD, USA) plug in (52). During RGC analysis, images were contrast adjusted, converted to 8 bit, and pseudo-colored red in the plug-in. Counts were averaged and normalized to mm^2^.

### 2.7 Optic nerve histopathology

Optic nerves were harvested and fixed in either 4% PFA for longitudinal histology or in 2% glutaraldehyde and 2% PFA for cross-sectional histology. The optic nerve for longitudinal histology was embedded in paraffin. Seven-micron-thick longitudinal sections were stained with Luxol Fast Blue (LFB)/H&E for assessment of demyelination using a 0-3 grading scheme with the following criteria: 0 = normal optic nerve, fully myelinated; 1 = scattered foci of demyelination; 2 = prominent foci of demyelination; and 3 = large areas of demyelination (53).

Seven-micron-thick longitudinal sections were deparaffinized in xylene and then rehydrated in a series of ethanol washes. Antigen retrieval was completed via exposure to boiling citrate buffer for 15 minutes. Samples were blocked with 1%BSA/0.3% TritonX-100/PBS solution for 2 hours at room temperature. The slides were then incubated with either rabbit anti-CD3 (ab135372 Abcam, Cambridge, United Kingdom), rabbit anti-Iba1 (019-19741 Wako Pure, Wako Chemicals, Richmond, VA, USA), or rabbit anti-Olig2 (ab136253, Abcam, Cambridge, United Kingdom), all diluted 1:250 in 1%BSA/0.3% TritonX-100/PBS overnight at 4°C. Slides were washed with PBS and then incubated with goat anti-rabbit Alexa Fluor 488 secondary antibody (ab150077 Abcam, Cambridge, United Kingdom, diluted 1:500 in 1%BSA/0.3% Triton-X100/1%/PBS) for three hours in the dark. The slides were rinsed again in PBS prior to incubation with DAPI (Roche Diagnostics GmBH, Mannheim, Germany, diluted 1:1000 in PBS) for 5 min before the coverslips were mounted with Aquamount (Epredia, Kalamazoo, Michigan, USA). For each optic nerve section, two representative images for immunohistochemical staining were taken at 20x, contrast was equally post-adjusted, and the numbers of CD3^+^, Iba1^+^, and Olig2^+^cells containing a DAPI^+^ nuclei were manually counted using ImageJ. The two values were averaged and normalized to mm^2^.

**Figure 3:**
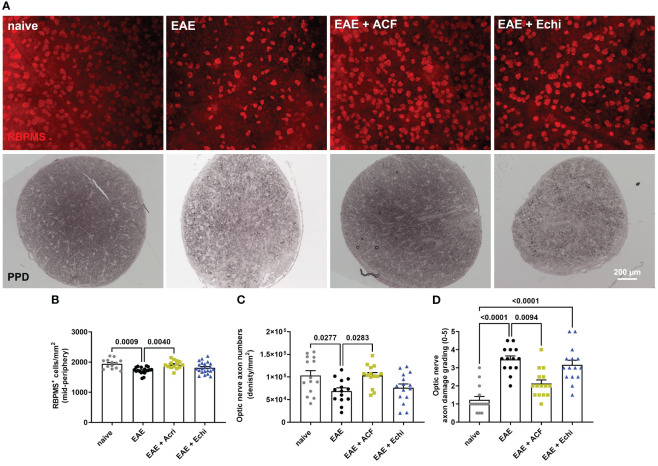
Effects of ACF treatment on RCG and optic nerve axon degeneration. **(A)** Representative images of RPBMS^+^ RCG density (upper panel) and PPD-stained optic nerve cross-sections from each EAE group indicate less degeneration in EAE+ACF mice. Analysis of RGC numbers **(B)** in the mid-peripheral retina, density of myelinated optic nerve axons **(C)**, and assessment of axon damage **(D)** confirmed protective effects of ACF in EAE mice. All data are given as mean ± SEM, with plots representing values of each retina. Differences between groups were calculated by one-way ANOVA with Tukey *post hoc* test, except for D), in which differences were calculated using the Kruskal–Wallis test.

For cross-sectional histology, the other optic nerve was processed as semi-thin sections using a modification of the TEM embedding protocol. Briefly, the optic nerve was fixed with 1% osmium, dehydrated in an ethanol series, penetrated with a series of propylene oxide (Sigma 110205)/Eponate 812 resin (18010, Ted Pella Inc., Redding, CA, USA), and embedded in Eponate 812 resin. Half-micron-thick sections were stained with 1% p-phenylenediamine (PPD; Sigma P6001). Optic nerve damage was graded by two investigators at 40x objective magnification on a Zeiss Axioscope 5 microscope in a masked fashion using a 0-5 grading scheme with the following criteria: grade 0 = healthy optic nerve; 1 = healthy nerve with only a few deeply stained (damaged) axons; 2 = slight damage with only few damaged axons; 3 = moderate damage with frequent degenerated axons; 4 = severe damage with mostly degenerated axons and the appearance of gliotic areas; 5 = severe axon degeneration in the optic nerve with frequent gliotic areas. Next, optic nerve axon density was determined in 8 micrographs per sample taken at 100x objective magnification in a cruciform pattern. Axon counts were completed in a semi-automatic manner using the ImageJ plugin AxoNet, as previously described (54). Briefly, TIFF images were opened in ImageJ, the area of interest was defined (off-target areas, if any, were excluded), the scale was set to 15.7 pixels/um, and the number of remaining myelinated axons was detected and counted by the plugin’s deep learning algorithm based on the U-Net-based encoder/decoder architecture (55). AxoNet has been shown to be robust in accounting for variations in tissue damage, image quality, and species of origin. Overlay images were visually examined for obvious discrepancies in the position and number of detected axons. Numbers of myelinated axons per image were normalized to the area and were displayed as optic nerve axon numbers relative to the area being sampled (density/mm^2^, **Figure 3C**).

### 2.8 Splenocyte activation assays

Murine splenocytes were isolated according to established protocols (56, 57). Briefly, following euthanasia, spleens were harvested and placed immediately into ice-cold HBSS (gibco 14175-095, Gibco by Invitrogen, Carlsbad, CA, USA) containing 1% pen/strep (gibco 15140-122). Spleens from each group were pooled together and mashed through a nylon cell strainer (Falcon 352340/352350, Corning Inc., Corning NY USA). Cells were then rinsed with 20ml DMEM-10 (DMEM, gibco 11965-092, with 10% FBS, gibco A31604-02, and 1% pen/strep), spun at 800x g, and incubated with 3ml RBC lysis buffer (Sigma R7757, Sigma-Aldrich, Inc., St Louis, MO, USA) on ice. Splenocytes were rinsed with 9ml DMEM-10, spun at 800x g, resuspended in 6ml DMEM-10 and counted using a Cellometer Auto T4 (Nexcelom, Waltham, MA, USA), and resuspended in an appropriate volume of RPMI-10 (RPMI, gibco 11875-095, with 10% FBS and 1% pen/strep). For all assays, spleens were pooled by group each day and run in triplicate.

For the MOG recall assay, EAE and control splenocytes were seeded in 96-well plates at 1.0x10^6^ cells per well. Cells from each group were then activated with 4.44ug/ml MOG (Hooke Laboratories, Lawrence, MA, USA) protein for 45 hours at 37C in 5% CO_2_ (https://hookelabs.com/protocols/cytokineProductionInVitro.html). Additional plates were seeded and treated in parallel for RNA collection for qPCR. At the end of the treatment phase, samples intended for RNA extraction were flash-frozen to -80C for future processing. Cell proliferation was then analyzed via a 3-(4,5-dimethylthiazol-2-yl)-2,5-diphenyl tetrazolium bromide (MTT) assay, as described in Roche Cell Proliferation Kit I (1 465 007 Roche Diagnostics, Laval, Quebec, Canada). Briefly, 10 ul of 5mg/ml MTT was added to all wells except a no-MTT control. The plate was then incubated for 3 hours prior to the addition of 100ul of solubilization solution (10%SDS in 0.01M HCl) at the 48hr timepoint, incubated overnight at 37C in 5% CO_2_, and read on a microplate reader at 570nm.

To determine ACF dose dependency, splenocytes from naïve or EAE mice were seeded in 96-well plates at 0.5x10^6^ cells per well. The cells were then activated with either 25ng/ml phorbol 12-myristate 13-acetate (PMA, Sigma P1585)/1ug/ml ionomycin (Iono, Sigma I9657), 50ul of 40ug/ml MOG_35-55_ (Hooke Labs DS-0111, Hooke Laboratories, LLC, Lawrence MA USA), or 2ul CD3/CD28 Dynabeads (Gibco 11456D) at 37C in 5% CO_2_ for 2-3 hours. The cells were then treated with either 1uM, 2uM, 4uM, or 6uM ACF after PMA/Iono activation or with 1uM, 2.5uM, 5uM, or 8.5uM ACF for the MOG recall assay and after CD3/28 activation, or they were left untreated for 21-22 hours at 37C in 5% CO_2_. At the end of the treatment phase, the samples intended for RNA extraction were flash-frozen to -80C for future processing, and proliferation in remaining samples was determined using an MTT assay.

### 2.9 RT-PCR

RNA was isolated from splenocytes using RNeasy columns (QIAGEN, Hilden, Germany). Isolated RNA was converted to cDNA using SuperScript Reserve Transcriptase (Thermo Fisher, Waltham, MA, USA) according to the manufacturer’s instructions using a SimpliAmp Thermal Cycler (Applied Biosystems, Waltham, MA, USA). Quantitative polymerase chain reaction (qPCR) was achieved with iTaq Universal Probes Supermix (BioRad, Hercules, CA, USA) through a QuantStudio3 (Applied Biosystems, Waltham, MA, USA) using the specific primers listed in **Table 1**. Amplification was normalized against that of the Tata Box-binding protein (*Tbp*). Expression changes were calculated using the ΔΔCT method.

**Table 1:** List of targets and primers.

Target	Forward	Reverse
*Atf4*	5’-CGTATTAGAGGCAGCAGTGC-3’	5’-AGGTATCTTTGTCCGTTACAGC-3’
*eIF2a*	5’-TCTCACCAAATAACACTGTCCT-3’	5’-CGATTTCAAACATGCTCCAGTT-3’
*Glut1*	5’-AGTTCGGCTATAACACTGGTG-3’	5’-GTGGTGAGTGTGGTGGATG-3’
*Hif-1α*	5’-GCTCACCATCAGTTATTTACGTG-3’	5’-CCGTCATCTGTTAGCACCAT-3’
*Vegf*	5’-CCGAAACCATGAACTTTCTGC-3’	5’-GACTTCTGCTCTCCTTCTGTC-3’
*Stat5a*	5’-TGCCCTCAACCTCACTACA-3’	5’-GCTCTCATCCAGGTCAAACTC-3’
*Tbp*	5’-TGTATCTACCGTGAATCTTGGC-3’	5’-CCAGAACTGAAAATCAACGCAG-3’

### 2.10 Statistics

All *in vivo* data were analyzed using the Shapiro–Wilk test. All numeric data that fulfilled the requirements of standard normal distribution were analyzed either using a one-way ANOVA followed by a Tukey *post hoc* test or by a two-way ANOVA followed by a Tukey *post hoc* test corrected for multiple measurements for spatial temporal analysis (EAE scoring). Data analysis of visual acuity, OCT, and pattern VEP as performed with a mixed-effect model using the maximum likelihood method to account for inter-eye differences and repeated measurements (left and right eye) on the same subject (58). Non-normally distributed data (Area under curve) and ordinal data for the grading of optic nerve demyelination and damage were analyzed using Kruskal–Wallis analysis followed by Dunn’s multiple comparison test. All results are given as mean ± standard error of the mean (SEM). Calculations were performed using GraphPad Prism, and p-values <0.05 were considered statistically significant. While all groups were included for statistical comparison, only significant differences between the cohorts are shown in the graphs.

## 3 Results

### 3.1 Motor–sensory impairment in EAE mice is mitigated by the HIF-1 inhibitor ACF

To determine the effect of two different HIF-1 inhibitors in EAE, we immunized 60 female C57BL/6J mice with MOG_33-55_, followed by systemic administration of either vehicle solution (PBS), ACF, or Echi (n=20/group). All immunized animals, regardless of their therapeutic intervention, developed an EAE typical of moderate-to-severe clinical manifestation over the time course of the study. The first symptoms were evident around day 12 post-immunization, with increasing severity in a caudal-to-rostral pattern. Peak of disease was reached at day 18–19, characterized by phenotypes ranging from partial hind limb paralysis (score of 2.5) to paralysis of one limb (score of 3). However, several EAE animals (n=5 in EAE, n=2 in EAE+ACF, and n=3 in EAE+Echi) were removed from the study either for humane reasons due to debilitating high EAE scores (4.5) or due to spontaneous death. The EAE animals that received systemic treatment with ACF starting on the day of EAE induction demonstrated a significantly milder disease course, whereas administration of Echi to EAE animals following the same treatment paradigm did not alter EAE progression (**Figures 1A, B**). We assessed visual acuity weekly as another sensitive functional outcome measurement and found that the pattern of visual impairment followed a similar time course as the clinical EAE signs. At day 14, a noticeable but statistically insignificant decline in visual acuity in all EAE animals was observed compared to the naïve mice. Subsequently, untreated and Echi-treated EAE mice continued to further decline to approximately half of their initial spatial frequency capability, with no recovery afterward. The slope of visual acuity decline in the EAE+ACF group was significantly milder; however, those EAE mice still experienced visual impairments and, again, with no noticeable improvement over time (**Figure 1C**). In summary, systemic administration of the HIF-1 inhibitor ACF, but not Echi, reduces the disease course and lessens the magnitude of ON-related visual decline in animals with MOG-induced EAE.

### 3.2 ACF treatment ameliorates RGC degeneration and positively affects optic nerve function

We next examined changes in the RGC/IPL complex architecture, which includes the RNFL, RCG, and IPL layer, by OCT as the degeneration of these retinal structures is usually related to disease progression in patients with MS and in EAE (29). All OCT image captures were centered at the optic nerve head, and none of the animals showed signs of adverse retinal phenotypes such as retinal detachments or uveitis. At 21 days post-immunization, by which point the EAE animals had already established significant visual impairment, only the EAE+Echi cohort revealed significant RGC/IPL complex thinning in comparison to the naïve controls and the EAE+ACF group (**Figure 2B**). Significant RGC/IPL complex thinning became evident in the untreated EAE mice, alongside further reduction in RGC/IPL complex thickness in the EAE+Echi group, at day 42 when compared to the naïve controls. These observations are in accordance with the decline in visual acuity in these two EAE cohorts. Analysis of EAE+ACF mice showed less pronounced, but still significant, retinal degeneration in comparison to the naïve controls. However, there was significant protection in contrast to the untreated EAE animals and the EAE+Echi group (**Figures 2A, B**). The partial protection of the retinal structure in the EAE+ACF cohort was consistent with their better visual acuity outcome.

To further characterize RGC and optic nerve function, we recorded pattern VEP at the end of the study as changes would indicate damage in the anterior visual pathway (59). Similar to the preservation of visual acuity and retinal structure, analysis of optic nerve conduction speed confirmed the beneficial effects of ACF in EAE mice. A total of 11% of waveforms were excluded from the analysis, of which 6% were due to absent pattern VEP signals. The other 5% related to adverse waveforms that did not follow the representative pattern VEP traces and did not contain the relevant peaks. The cumulative pattern VEP trace overlays from each group depicted a delayed N1 trough and significantly prolonged latency of the P2 peak in all EAE groups. Interestingly, while the EAE and EAE+Echi groups also showed a significantly decreased amplitude, the signal strength of the EAE+ACF cohort almost exceeded the amplitude of the naïve controls. We found a higher variability in the EAE+ACF cohort and measured a twofold higher amplitude than in the controls in some animals of this group (**Figures 2C, D**). While the mean signal strength of the controls and EAE+ACF mice were similar, both the averaged N1 amplitudes and the N1-to-P2 amplitudes in the EAE group and in the EAE+Echi cohort were significantly declined. Although both HIF-1 inhibitors were unable to prevent the delay in optic nerve conduction speed, the preservation of the amplitude found in the EAE+ACF group suggests that ACF treatment influences the electrical activity of RGC in EAE mice.

To validate our *in vivo* results, we harvested eyes and optic nerves 42 days after EAE induction. We first evaluated RGC survival by RBPMS immunolabeling of the retinal wholemounts. Analysis of RGC density demonstrated the preservation of RBPMS^+^ RGC in the retinas of the EAE+ACF cohort, which showed RGC numbers similar to the controls. As expected, we observed significant RGC loss in the untreated EAE mice and an insignificant but noticeable reduction of RGC in the EAE+Echi group (**Figures 3A, B**). The findings of mitigated RGC degeneration in the EAE+ACF group were further confirmed through the analysis of optic nerve axon density and assessment of optic nerve damage. One optic nerve per animal underwent PPD staining to determine the number of remaining myelinated axons, as these correspond to the numbers of RGC, and degeneration based on a histopathologic grading scheme. Similar to our OCT data and RGC results, we found significantly decreased numbers of myelinated axons in the untreated EAE group when compared to both the controls and the EAE+ACF cohort. A reduction of optic nerve axon density was observed in the EAE+Echi group, but this did not reach statistical significance (**Figures 3A, C**). Subsequent grading, which focuses on the histopathological assessment of amounts of degenerating axons, myelin loss, and areas of gliotic scar formation, confirmed that the untreated EAE group demonstrated significantly higher grades of degeneration. A similar trend was also observed in the EAE+Echi cohort, which exhibited significant optic nerve degeneration when compared to the naïve controls. Along with the findings of preserved RGC structure and function in EAE+ACF animals, we observed lower grades of degeneration (less degeneration) in this group (**Figures 3B, D**). In summary, RCG and optic nerve axon analysis demonstrated that ACF treatment in the EAE animal model showed evidence of structural preservation that coincides with our results showing the preservation of neuronal function.

### 3.3 ACF treatment suppresses optic nerve inflammation and myelin degeneration

**Figure 4:**
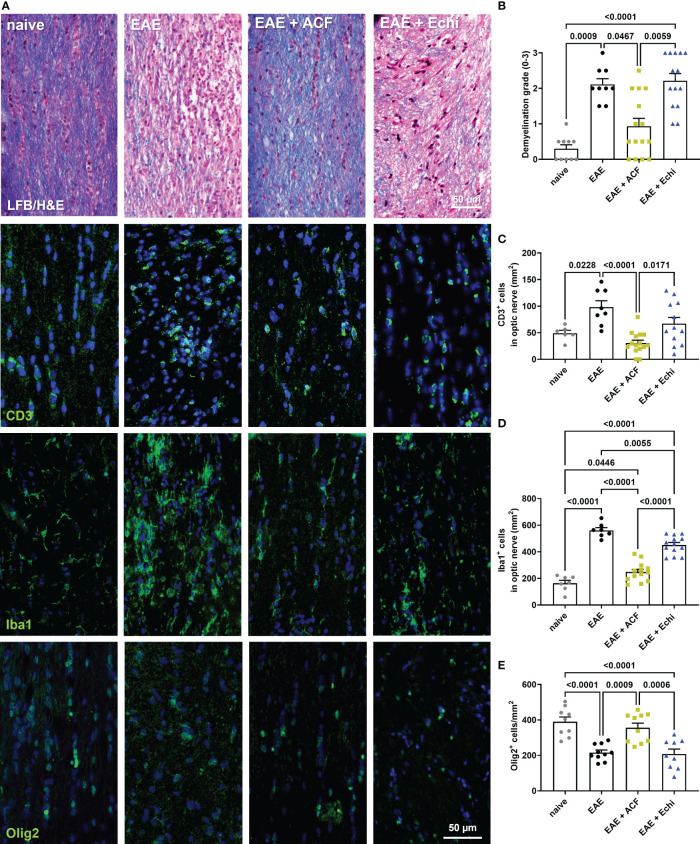
Optic nerve immunohistopathology 42 days after EAE induction. **(A)** Representative images of longitudinal optic nerve sections from naïve controls and EAE mice stained with LFB/H&E and for assessment of CD3^+^ cells, Iba1^+^ microglia/macrophages, and Olig2^+^ oligodendrocytes. **(B)** Status of demyelination grading revealed protection of myelination in EAE+ACF mice, which was accompanied by reduced CD3 cell infiltration **(C)**, decreased microglia activation **(D)**, and increased numbers of Olig2^+^ cells **(E)** when compared to untreated EAE mice and the EAE+Echi cohort. Statistics for graph in C were calculated by non-parametric test using the Kruskal–Wallis test; all other data were determined by ANOVA and Tukey *post hoc* test.

To gain more insights into optic nerve histopathology, we determined the status of myelination and inflammation in longitudinal sections of the remaining contralateral optic nerves (**Figures 4**). LFB/H&E-stained optic nerve sections obtained from the untreated EAE mice and the EAE+Echi group both showed clear evidence of demyelination and increased amounts of infiltrated cells when compared to the naïve controls or the EAE+ACF group. In the latter group, only signs of some minor demyelination were detected, accompanied by a few scattered cells. Immunostaining for CD3 and Iba1 further confirmed a significantly improved ON phenotype in the EAE+ACF mice compared to a prominent T cell infiltration and severe microglia/macrophage activation, especially in the untreated EAE cohort (**Figures 4B–D**). In accordance with the reduced demyelination in the EAE+ACF group, the number of Olig2^+^ cells was found to be similarly high to that of the controls. A significant reduction to about half of the Olig2^+^ cells amount was observed in the untreated EAE and EAE+Echi group (**Figures 4A, E**). These data coincide with our results showing an improved phenotype of visual structure and function in EAE animals having received ACF treatment. EAE mice that were treated with Echi, the HIF-1 inhibitor more specific to VEGF signaling, demonstrated minor improvement in some outcome parameters but overall did not seem to demonstrate significant protective effects.

### 3.4 ACF facilitates immunomodulatory properties

**Figure 5:**
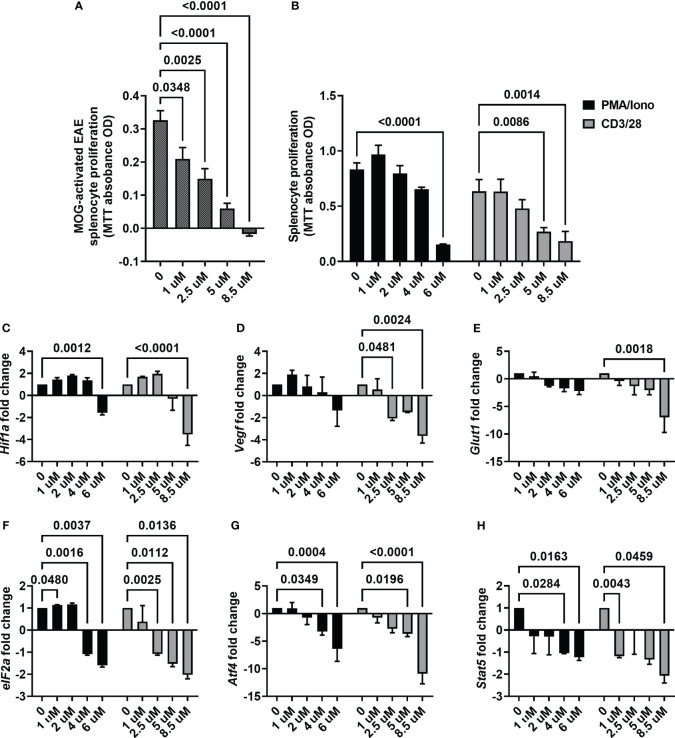
ACF immunomodulatory properties on activated splenocytes. **(A)** ACF demonstrated a dose-dependent decrease in the proliferation of splenocytes from EAE mice that were restimulated with MOG_33-55_ (shaded gray bars) and similar immunomodulatory effects on PMA/Iono (black bars)- or CD3/28 beads (gray bars)-stimulated naïve splenocytes **(B)**. Examination of changes in HIF-1 pathway-relevant genes revealed target engagement in stimulated naïve splenocytes with a dose-dependent downregulation of *Hif1-a* **(C)**, *Vegf* **(D)**, and *Glut1* **(E)** expression. Furthermore, ACF facilitated blocking of UPR, evident by the downregulation of *eIF2a* **(F)** and *Atf4* **(G)** expression, and negatively affected *Stat5* expression in a dose-dependent manner **(H)**.

With the observation of ACF being effective in mitigating motor and sensory impairment, we next focused on its mechanism of action. First, we determined the proliferation rate of splenocytes harvested from the EAE mice upon *ex vivo* re-exposure to MOG, which were then treated with different ACF concentrations. We observed that ascending concentrations of ACF led to a significant reduction of proliferation in a dose-dependent manner when compared to the untreated MOG-activated EAE splenocytes. Of note, EAE splenocyte proliferation was completely blocked at the highest concentration of 8.5 uM ACF (**Figure 5**). Next, we confirmed ACF dose dependency in PMA/Iono- and/or CD3/28-stimulated naïve splenocytes. Again, treatment with 6 uM ACF significantly decreased PMA/Iono-activated splenocyte proliferation, and both higher dosages (5 and 8.5 uM ACF) demonstrated significant immunomodulatory properties in splenocytes after CD3/28 stimulation (**Figure 5B**). To validate the engagement of HIF-1-relevant pathways and with a focus on the Treg/Th17 balance and eIF2a/ATF4 UPR axis, we conducted RT qPCR on relevant gene markers. Expression of *Hif-1α* itself demonstrated a noticeable, albeit insignificant, increased expression at 1 and 2.5 uM ACF but was found to be significantly decreased after exposure to 8.5 mM ACF (**Figure 5C**). Our data further indicated that ACF had strong inhibitory effects on *Vegf* and *Glut1* expression in CD3/28-activated splenocytes but not on PMA/Iono stimulation (**Figures 5D, E**). In PMA/Iono-stimulated splenocytes, ACF treatment primarily induced a slight, up to 2-fold, increase in *Vegf* expression followed by a dose-dependent decline. With respect to the UPR on the eIF2a/ATF4 axis, expression for both genes was found to be significantly downregulated at higher ACF dosages. The strongest effect of ACF was noticed on *Atf4* expression, with a more than 10-fold decrease at 8.5 uM after CD3/28 stimulation (**Figures 5F, G**). A similar trend of a statistically significant down-regulated expression in activated splenocytes after ACF exposure was determined for *Stat5* (**Figure 5H**). In summary, our data suggest that ACF not only inhibits the expression of HIF-1 downstream genes, such as *Vegf* and *Glut1*, but also has a strong inhibitory effect on UPR signaling. However, these results needed to be interpreted in view of a globally dose-dependent decrease in proliferation.

## 4 Discussion

Immunomodulation is the most common treatment approach for managing MS, and disease-modifying therapies (DMTs) are effective at prolonging the time between demyelinating events. However, most DMTs fail to facilitate neuroprotection or remyelination and thus are ineffective in reversing MS symptoms. Here, we report preclinical data on a previously FDA-approved drug, ACF, on immunomodulatory and, more importantly, potentially beneficial effects on visual function and structure in the EAE mouse model of MS.

In accordance with previously published studies (29, 37–39), our MOG-induced EAE model mounts a well-characterized ON with many clinically relevant outcomes such as acute vision loss, thinning of the RGC/IPL complex and loss of RGC, and changes in the pattern VEP. These attributes make this model extremely valuable to determine new treatment options to supplement DMT in order to prevent vision loss, as visual impairment has been recognized as one of the most common causes of decrease in quality of life in MS (60). We were able to demonstrate that systemic administration of ACF mitigates motor and sensory impairments, as evidenced by the significantly less severe degeneration of visual function and structure. However, ACF treatment at the used dosage does not prevent mice from developing EAE but does significantly mitigate the clinical severity. Consistent with this observation is the less severe, but still significant, impairment in visual acuity as a result of a milder phenotype of optic neuritis in ACF-treated EAE mice. Because of the nature of the EAE model in which the pathology is clearly immune cell driven, it is difficult to conclude potential neuroprotective effects of drug compounds that also have immunomodulatory properties. The preservation of function and structure in the visual system could simply be just the logical consequence of decreasing immune cell-related demyelination and subsequent neuronal loss. This statement also applies to the results of our study, and we show that ACF has strong antiproliferative effects on stimulated splenocytes. However, there is also evidence for restorative processes in our EAE study. Firstly, the pattern VEP waveforms in our EAE+ACF group not only displayed a preserved amplitude but also a wide N1 trough. The amplitude in this group was found to be slightly higher than the naïve controls, which might be the result of ACF increasing the electrical activity of RGC. This enhanced electrical input could explain the wide N1 trough and subsequently led to the increase in conduction speed. Similar observations of a slightly abnormal but preserved VEP waveform have been reported in an EAE study after RGC-specific knockout of *eIF2a*. Here, eIF2a inhibition further provided the preservation of RGC function and optic nerve structure (61). Secondly, the increased numbers of Olig2-positive cells in the optic nerve of ACF-treated EAE mice indicates a potential beneficial effect of ACF, which can result in increased remyelination. A recent publication has demonstrated that chronic HIF-1α accumulation in pluripotent stem cell-derived oligodendrocyte progenitors blocked differentiation. Inhibition of HIF-1 signaling restored oligodendrocyte formation even in partially hypoxic human oligocortical spheroids (62). Both observations fit well with the theory that demyelinated axons that are electrically intact with properly firing neurons provide an increased likelihood for remyelination [reviewed in (63, 64)]. This effect might also be related to the ability of ACF to block HIF-1 signaling in oligodendrocyte precursor cells, which further benefits remyelination or at least might prevent Olig2^+^cells from dying in the EAE model of MS. However, our second argument would benefit from more supportive data, such as confirmation via g-ratio histological analysis of optic nerve transverse sections to show increases in remyelination and through additional *in vivo* experiments. Potentially, in future studies, animal models of toxin-induced demyelination and optic nerve transection/crush might be suitable to determine whether ACF facilitates remyelination and neuroprotection. The involvement of the immune system in both of these *in vivo* models of optic nerve injury still needs to be considered, but it is arguably not the major driving factor for demyelination as in the EAE model.

Undoubtably, ACF decreases cell proliferation, as recent cancer-related research studies have demonstrated (41, 42). ACF also affects UPR, an essential mechanism for maintaining cell homeostasis and survival. Activation of the UPR pathway, caused by several stressors including hypoxia and pro-inflammatory condition, can lead to apoptosis in cells via the ATF4/CHOP axis (65), and it also determines the fate of T cell differentiation [reviewed in (66, 67)]. More precisely, ATF4 is important for T cells in that it promotes the proliferation of activated CD4+ T cells through metabolic reprogramming. ATF4-deficient mice have a reduced Th1 profile but elicit an increased Th17 response, leading to an exacerbated experimental allergic encephalomyelitis (68). With respect to UPR, a recent publication showed that ACF blocks eIF2a phosphorylation, subsequently blocking ATF4 transcription (41). In a study using skin fibroblasts from MS patients, it was observed that there was increased gene expression in the ATF4/CHOP axis (69), leading us to speculate that the upregulation of ATF4 and eIf2a is an important contributor to MS. ACF does not alter HIF-1 protein expression (70), and thus, we conclude that the decrease in Hif1α transcription at 8.5µM ACF treatment could be the result of a global decrease in gene expression. Just as we found a decrease in *Atf4* expression in a dose-dependent manner, we also found a decrease in *Stat5* expression. STAT5 is another vital transcription factor in the differentiation of T cells. Specifically, STAT5 causes the differentiation into Tregs and Th1 cells while blocking the generation of Th17 cells (71). While these findings slightly contradict our study, it is possible that levels of other T cell subtypes are also reduced upon ACF treatment, and thus, a global decrease of immune cells facilitates the protective effect in our EAE model. Future work needs to be done to determine Treg and Th17 balance in more detail using immunophenotyping approaches for both *in vivo* and *in vitro* assays on EAE+ACF vs EAE and splenocyte proliferation in our MOG recall assay.

Our study demonstrated that ACF administration mitigated both visual and motor–sensory deficits. However, our study contains some limitations; for instance, we only determined the effects of a single dose of ACF and one treatment regimen of ACF *in vivo* as a proof-of-concept study. The *ex vivo* splenocyte stimulation experiments indicated a clear dose–response effect. Despite a positive outcome (protective effects on CNS and visual system function and structure), a potential future study design should, therefore, include various treatment regimens, different routes of administration, and varying concentrations of ACF to increase the translational rigor. The listed echinomycin dosage of 10µg/kg has been used in previous studies (49); however, our results do not indicate beneficial treatment in an EAE model. Another limitation in our study is that the treatment was started at the time of induction, which is a suitable proof-of-concept design, but it does not relate to the clinical circumstances of acute optic neuritis treatment where patients receive an interventional treatment days after symptom onset, not prophylactic therapy. A significant strength of our study is the use of highly translational readouts of visual function and structure. Our parameters have been adapted from clinical practice to measure visual acuity, record optic nerve electrophysiology, and image the RGC/IPL complex using OCT. Another important aspect of our study is that ACF is the most potent HIF-1 inhibitor that is already approved by the FDA. Therefore, the repurposing of the drug and implementation into the clinic, as seen for various diseases such as cancer (72), are highly probable. As previously mentioned, our study showed proof of concept with ACF reducing visual impairments and motor–sensory deficits in a mouse model relevant to MS. We also demonstrated even stronger inhibitory effects on eIF2α and ATF4 expression in comparison to HIF-1 related markers, which has been unappreciated by previous literature. However, regulation of relevant gene markers was only analyzed on the RNA level, and future studies should corroborate these findings at the protein level. Previous publications have shown that topically applied ACF can be absorbed into the retina and exert its effects (45). Therefore, a suitable route for the administration of ACF in the treatment of optic neuropathies might be topical administration using eye drops. Because of the autofluorescent properties of ACF, it is also feasible to track its distribution in the retina *in vivo*, which would allow for further determination of ACF bioavailability and its mechanism of action in the CNS, specifically in the visual system. In summary, the unique and specific properties of ACF make this compound an attractive drug candidate for MS and also for the treatment of eye diseases where inhibition of Hif-1 signaling and blocking of the UPR are desirable.

Altogether, our findings demonstrated that ACF successfully reduced motor–sensory impairment and preserved visual function along with structure of the visual pathway. Current DMTs used in MS therapies prolong the timeframe between relapses but do not provide any opportunity to reverse the progression of vision loss in regenerative terms. Here, we have presented ACF as a possible therapeutic for the rehabilitation of MS-induced ON by inhibiting the HIF-1 pathway and UPR. ACF has beneficial pleiotropic effects on both immunomodulation and cell stress response, making it a strong candidate for future clinical implementations either to be coupled with current DMTs or potentially as a new standalone therapy.

## Data availability statement

The original contributions presented in the study are included in the article/supplementary material. Further inquiries can be directed to the corresponding author.

## Ethics statement

The animal study was approved by Iowa City VA Health Care Systems IACUC. The study was conducted in accordance with the local legislation and institutional requirements.

## Author contributions

OG: Conceptualization, Data curation, Formal Analysis, Investigation, Methodology, Resources, Supervision, Visualization, Writing – original draft, Writing – review & editing. JA: Conceptualization, Data curation, Investigation, Visualization, Writing – original draft. BE: Formal Analysis, Investigation, Methodology, Validation, Visualization, Writing – original draft, Writing – review & editing. RK: Formal Analysis, Funding acquisition, Writing – review & editing.
